# Understanding the rapid spread of antimicrobial resistance genes mediated by IS*26*

**DOI:** 10.1002/mlf2.12114

**Published:** 2024-03-18

**Authors:** Na Tang, Dawei Wei, Yuan Zeng, Gang Zhang, Chao Wang, Jie Feng, Yuqin Song

**Affiliations:** ^1^ State Key Laboratory of Microbial Resources, Institute of Microbiology Chinese Academy of Sciences Beijing China; ^2^ College of Life Science University of Chinese Academy of Sciences Beijing China

**Keywords:** antibiotic consumption, antibiotic resistance genes, horizontal gene transfer, insertion sequence, IS*26*

## Abstract

Insertion sequences (ISs) promote the transmission of antimicrobial resistance genes (ARGs) across bacterial populations. However, their contributions and dynamics during the transmission of resistance remain unclear. In this study, we selected IS*26* as a representative transposable element to decipher the relationship between ISs and ARGs and to investigate their transfer features and transmission trends. We retrieved 2656 translocatable IS *26* ‐bounded units with ARGs (tIS*26*‐bUs‐ARGs) in complete bacterial genomes from the NCBI RefSeq database. In total, 124 ARGs spanning 12 classes of antibiotics were detected, and the average contribution rate of IS*26* to these genes was 41.2%. We found that IS *26* ‐bounded units (IS*26*‐bUs) mediated extensive ARG dissemination within the bacteria of the *Gammaproteobacteria* class, showing strong transfer potential between strains, species, and even phyla. The IS*26*‐bUs expanded in bacterial populations over time, and their temporal expansion trend was significantly correlated with antibiotic usage. This wide dissemination could be due to the nonspecific target site preference of IS*26*. Finally, we experimentally confirmed that the introduction of a single copy of IS*26* could lead to the formation of a composite transposon mediating the transmission of “passenger” genes. These observations extend our knowledge of the IS*26* and provide new insights into the mediating role of ISs in the dissemination of antibiotic resistance.

## Impact statement

IS*26*‐mediated drug resistance gene transmission has been increasingly reported. However, a comprehensive understanding of IS*26* contribution to the spread of drug resistance is lacking. Our study demonstrated the unexpectedly high distribution in bacteria, especially the *Gammaproteobacteria* class, as well as the significant contribution of IS*26*‐bounded units (IS*26*‐bUs) in mediating the interspecies and even interphyla dissemination of ARGs among bacteria. Notably, the expansion of IS*26*‐bUs exhibited a temporal trend. Nonspecific target site preference could be one main mechanism for the rapid expansion of IS*26*‐bUs. Our study provided new insights into the comprehensive understanding of the relationship between ISs and ARGs.

## INTRODUCTION

Bacterial antimicrobial resistance is a serious worldwide public health problem. Antimicrobial resistance genes (ARGs) are usually associated with mobile genetic elements, and the sequencing of bacterial genomes has revealed a large repertoire of resistance determinants located on various transposable elements, including plasmids, insertion sequences (ISs), transposons, integrons, and integrative conjugative elements^1^, ^2^. These mobile genetic elements promote the acquisition and transmission of ARGs, as they play a vital role in facilitating horizontal gene transfer within or between DNA molecules. ISs are generally small mobile elements encoding the enzymes necessary for their transposition; they are capable of repeated insertion into many different sites within a genome^3^. Moreover, ISs are known to mediate the mobilization of ARGs as part of a composite transposon, that is, an element with two bounded copies of the same or related IS moving as a single unit^4^. A recent study also demonstrated that ISs could interact with conjugative plasmids to influence the horizontal transfer of ARGs^5^. Therefore, ISs are considered to contribute significantly to the spread of ARGs. However, we still lack the knowledge required to evaluate the ARG content, phylogenetic distribution, and temporal dynamics of specific ISs.

IS*26*, a member of the IS*6* family, is frequently detected in a variety of clinically resistant bacterial isolates and has attracted considerable attention due to its contribution to the spread of antibiotic resistance. Upon insertion, IS*26* generates short 8‐bp directly repeated (DR) sequences of the target DNA flanking the inserted IS*26*‐based element^6^. Transposition, based on either a replicative or conservative mechanism, may lead to the formation of an IS*26*‐bounded composite transposon comprising two IS*26* elements flanking a central DNA segment. This element has been widely reported to be closely genetically related to various ARGs. In the year 1982, an IS*26*‐based transposon, Tn*2680*, was first reported to mediate the kanamycin resistance^7^. Thereafter, IS*26*‐bounded transposons have been increasingly reported on multidrug‐resistant plasmids from clinical bacterial isolates in association with multiple ARGs, such as *aphA1*, *tetM*, *bla*_CTX‐M‐55_, *bla*_NDM_, and *bla*_KPC_ ^8^, ^9^, ^10^, ^11^, suggesting a role of IS*26* in the transmission of these resistance genes. Furthermore, our previous study indicated that IS*26* could mediate rapid amplification of *bla*_KPC_ and increase carbapenem resistance^12^, which has a significant clinical impact.

In this study, we selected IS*26*, a crucial mobile element for the dissemination of antimicrobial resistance, to understand the IS‐mediated rapid spread of ARGs. We performed a comprehensive analysis of IS*26* and IS *26* ‐bounded units (IS*26*‐bUs) in complete bacterial genomes from the NCBI RefSeq database, explored the relationships among IS*26*, ARGs, and antibiotic consumption, evaluated the potential of horizontal gene transfer of IS*26*, and analyzed the temporal trend of IS*26* dissemination in bacterial populations. This study reveals a significant contribution of the IS*26* element to the dissemination of prevalent ARGs in *Gammaproteobacteria*, proposes reasons for the frequent transfer, and deepens our understanding of the contribution of ISs to the spread of ARGs.

## RESULTS

### Prevalent ARG types are enriched in tIS*26*‐bUs

**Figure 1:**
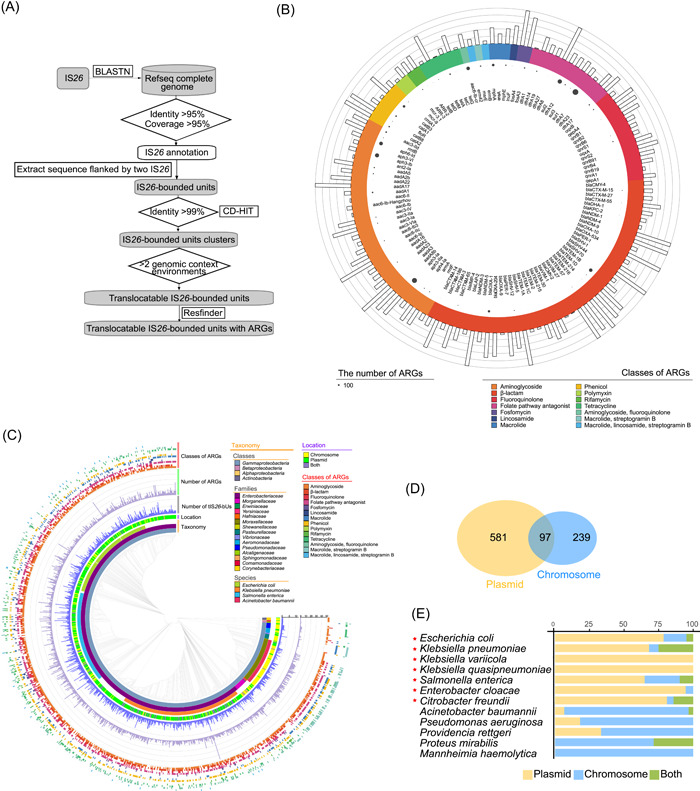
Construction and characterization of tIS*26*‐bUs‐ARGs. (A) The technical flow of the construction of the tIS*26*‐bUs‐ARGs data set. (B) Distribution of ARGs carried by tIS*26*‐bUs. From inner to outer, the genes in the first ring indicate the ARGs carried by tIS*26*‐bUs, the size of the circles in the second ring indicate the number of ARGs, the different colors in the third ring indicate the antibiotic classes for which resistance is conferred by the annotated ARGs, and the histograms in the fourth ring indicate the contribution rate of tIS*26*‐bUs to ARGs. (C) The phylogenetic tree, constructed based on the genomes of the tIS*26*‐bUs‐ARGs‐positive strains. From inner to outer, the first three rings indicate the taxonomy of each strain. The fourth ring shows the location of tIS*26*‐bUs‐ARGs (yellow, chromosome; green, plasmid; purple, both chromosome and plasmid). The fifth ring shows the number of tIS*26*‐bUs‐ARGs detected on each strain. The sixth ring shows the number of ARGs carried by each strain. The seventh ring shows the classes of the ARGs carried by each strain. (D) Venn diagram shows the relationship between the strain sets of plasmid‐located tIS*26*‐bUs‐ARGs and that of chromosome‐located tIS*26*‐bUs‐ARGs. (E) The distribution of plasmid‐ and chromosome‐located tIS*26*‐bUs‐ARGs of strains from different species. The red stars indicate the species of *Enterobacteriaceae*. ARGs, antimicrobial resistance genes; tIS*26*‐bUs, translocatable IS*26*‐bounded units; tIS*26*‐bU‐ARGs, translocatable IS*26*‐bounded units with antimicrobial resistance genes.

To explore the role of IS*26*‐bUs in the dissemination of antibiotic resistance, we established a comprehensive data set of 2656 translocatable IS*26*‐bUs with ARGs (tIS*26*‐bUs‐ARGs) (Figure 1A) belonging to 340 clusters (Table S1) based on 12,142 high‐quality complete bacterial genomes of NCBI RefSeq genome database.

A total of 5439 ARGs belonging to 124 ARG types that confer resistance to 12 classes of antibiotics were detected in the tIS*26*‐bUs. All of the antibiotic classes encompassed broad‐spectrum antibiotics widely used in humans and animals. None of the identified ARGs confer resistance to antibiotics that specifically target Gram‐positive bacteria, indicating that the tIS*26*‐bU mainly mediated ARG spread in Gram‐negative bacteria (Figure 1B). β‐lactam is one of the most widely used classes of antibiotics worldwide; accordingly, β‐lactam ARG types were detected most frequently (42) in the tIS*26*‐bU. Aminoglycoside resistance genes (31), fluoroquinolone resistance genes (13), and folate pathway antagonist resistance genes (12) were also prominent, while there were <10 ARGs that confer resistance to other antibiotics (such as tetracycline, macrolide, and polymyxin). The contribution rates of tIS*26*‐bUs to the 124 ARG types ranged from 1.6% to 100% (Figure 1B). The average contribution rate was 41.2%, with a standard deviation of 2.6%, highlighting the role of IS*26* in the dissemination of antibiotic resistance. Importantly, some variants of the New Delhi metallo‐β‐lactamase and extended‐spectrum β‐lactamase genes, such as *bla*_NDM‐3_, *bla*_NDM‐4_, *bla*_NDM‐9_, *bla*_SHV‐5_, *bla*_SHV‐44_, *bla*_TEM‐216_, *bla*_OXA‐534_, and *bla*_VIM‐27_, which have emerged as severe threats, were located only on tIS*26*‐bUs, indicating that IS*26* is a unique shuttle bus for these ARGs. Specifically, tIS*26*‐bUs commonly carry 1–10 ARGs (average of 2.05).

### tIS*26*‐bUs mediate extensive ARG dissemination within the *Gammaproteobacteria*

To understand the extent to which tIS*26*‐bUs mediate the dissemination of ARGs, we constructed a phylogenetic tree to analyze the phylogenetic relationships among the known hosts of this element (Figure 1C). Overall, 2651 tIS*26*‐bUs‐ARGs were found in 69 species of *Proteobacteria*, and five were found within four species of the phylum *Actinobacteria*. The majority (2644, 99.7%) of tIS*26*‐bUs were distributed within 64 species of *Gammaproteobacteria*; only one and six tIS*26*‐bUs were identified in *Alphaproteobacteria* and *Betaproteobacteria*, respectively (Figures 1C and S1). These results indicate that *Gammaproteobacteria* is the main host of tIS*26*‐bUs‐ARGs, and that these units are capable of crossing phylum barriers, even between Gram‐positive and ‐negative bacteria. Among all of the species analyzed, four were identified as highly abundant carriers of tIS*26*‐bUs; all these four species are clinically notorious pathogens: *Escherichia coli*, *Klebsiella pneumoniae*, *Salmonella enterica*, and *Acinetobacter baumannii* (Figure 1C). Furthermore, we found that one strain could carry 1–12 clusters of tIS*26*‐bUs‐ARGs and up to 48 ARGs conferring resistance to 10 classes of antibiotic (Figure 1C), suggesting the complexity of resistant bacteria and the high risk of antibiotic resistance mediated by tIS*26*‐bUs‐ARGs. Moreover, more than half (581/917 = 63.3%) of the strains carried tIS*26*‐bUs‐ARGs only on the plasmids, 26.1% of the strains carried tIS*26*‐bUs‐ARGs only on the chromosomes, while a small proportion (10.6%) of the strains carried both plasmid‐ and chromosome‐located tIS*26*‐bUs‐ARGs (Figure 1D). It was interesting that the location of the tIS*26*‐bUs‐ARGs showed taxonomy preference among the strains. The tIS*26*‐bUs‐ARGs were mainly located on plasmids in strains of *Enterobacteriaceae*, while they were more likely to be located on chromosomes in strains of non‐*Enterobacteriaceae*, like *A. baumannii* (Figure 1E).

**Figure 2:**
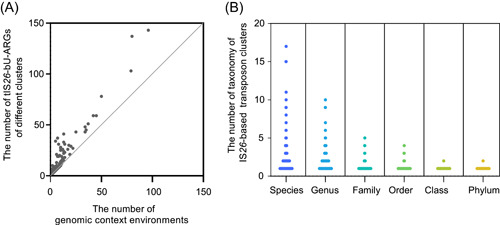
Transfer capacity of the tIS*26*‐bUs‐ARGs. (A) The result of the genomic context environments of tIS*26*‐bUs‐ARGs in the same cluster. (B) The result of the distribution of tIS*26*‐bUs‐ARGs in the same cluster at different taxonomic levels.

To evaluate the transfer capacity of the tIS*26*‐bUs, we analyzed the genomic context environments of the 340 clusters. These clusters were inserted into 2–96 different genomic environments (mean of 5.42; standard deviation of 9.76) (Figure 2A) and identified in 1–17 species, 1–10 genera, 1–5 families, 1–4 orders, 1–2 classes, and 1–2 phyla (Figure 2B). This implies that tIS*26*‐bUs are frequently transferred, including across phylogenetically distant bacteria. The most frequent tIS*26*‐bU cluster is Cluster2630, consisting of an *aph*(3′)‐Ia gene flanked by two IS*26*s (Figure S2A). A total of 143 tIS*26*‐bUs were identified in Cluster2630; they were located in 96 different genomic environments and distributed among 17 species within *Gammaproteobacteria* (Table S1); 101 tIS*26*‐bUs were located on plasmids, and 42 were located on chromosomes. Overall, the tIS*26*‐bUs within Cluster2630 demonstrated its high capacity to disseminate among bacteria. Cluster2041, carrying the carbapenem resistance gene *bla*_KPC‐2_ (Figure S2B), was found in nine different genomic environments in *K. pneumoniae*, suggesting frequent transfer of this transposon and a high risk of spreading of carbapenem‐resistant genes by tIS*26*‐bUs. Cluster2557 carrying an *aph*(3′)‐Ia and a transcriptional regulator (Figure S2C) was found in the Gram‐positive bacteria phylum *Actinobacteria*, as well as in six species, four genera, three families, two orders, and one class of the phylum *Proteobacteria*, indicating high ability of the transposon to facilitate horizontal gene transfer of the aminoglycoside resistance gene across phylogenetically distant bacteria.

### Expansion of tIS*26*‐bUs‐ARGs in bacterial populations over time

**Figure 3:**
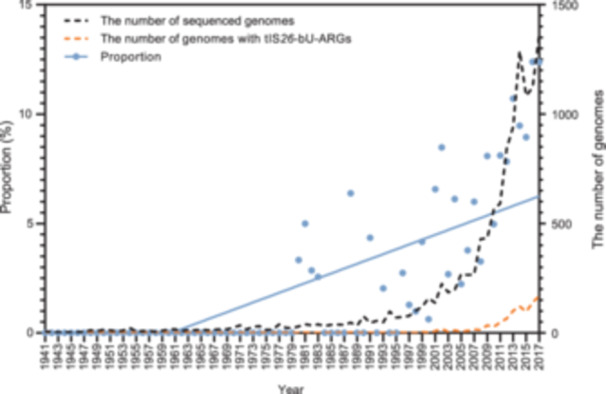
The scatter plots of the proportion of tIS*26*‐bUs‐ARGs positive strains in sequenced strains over time.

To understand the temporal dynamics of tIS*26*‐bUs, we calculated the proportion of tIS*26*‐bUs‐ARG‐positive genomes in sequenced strains over time. The obtained linear regression equation, *Y* = 0.001103 *X* – 2.162 (*R* ^2^ = 0.54, *p* < 0.0001), indicated a trend toward expansion of tIS*26*‐bUs. From 2005 to 2017, the proportion of genomes with tIS*26*‐bUs‐ARGs increased steadily. The number of tIS*26*‐bUs‐ARG‐positive strains increased from 6 to 169, and the proportion of tIS*26*‐bUs‐ARG‐positive strains in sequenced strains rose from 3.2% to 12.4% (Figure 3). This result demonstrates the expansion of tIS*26*‐bUs‐ARGs among bacterial populations over time. The Cochran–Armitage and the Mann–Kendall tests were performed to determine the temporal trend of the strains with tIS*26*‐bUs‐ARGs. The *p *< 0.0001 convincingly indicated that the proportion of tIS*26*‐bUs‐ARG‐positive strains within the sequenced strains followed a monotonic increasing trend. We also analyzed the dynamics of tIS*26*‐bUs‐ARGs from different sources, including human‐, animal‐, and environment‐derived strains. The scatter plots and Mann–Kendall test results showed that the proportion of tIS*26*‐bUs‐ARG‐positive strains also followed a monotonic increasing trend across all three sources (Figure S3A and Table S2). We confirmed this dynamic trend by conducting random sampling, which clearly showed that the tIS*26*‐bUs‐ARGs disseminated among bacterial populations over time (Figure S3B and Table S2). Additionally, we compared the proportion of tIS*26*‐bUs‐ARG‐positive strains from different sources. There was no significant difference between the proportions of tIS*26*‐bUs‐ARG‐positive strains in human‐ and animal‐origin strains, while the proportions of human‐origin were significantly higher than those of environmental‐origin (Figure S3C), suggesting the higher effect of human and animal than environment onto the tIS*26*‐bUs‐ARG‐positive strains.

The host of tIS*26*‐bUs‐ARGs also changed over time. From 1980 to 1989, tIS*26*‐bUs‐ARGs were detected in only five species of *Gammaproteobacteria*. From 1990 to 1999, tIS*26*‐bUs‐ARGs were not only distributed in two additional species of *Gammaproteobacteria*, but also in one species of *Actinomycetia*. From 2000 to 2009, the host range of tIS*26*‐bUs‐ARGs increased by seven species of *Gammaproteobacteria*; and from 2010 to 2017, tIS*26*‐bUs‐ARGs spread to additional 51 species of *Gammaproteobacteria*, four species of *Betaproteobacteria*, one species of *Alphaproteobacteria*, and three species of *Actinomycetia* (Table S3).

### The spread of β‐lactam resistance mediated by IS*26*‐bUs correlates with antibiotic usage

As β‐lactam ARG types are most abundant in the IS*26*‐bUs, we performed a correlation analysis of the relationship between tIS*26*‐bUs carrying β‐lactam resistance genes (tIS*26*‐bU^β‐lactam^) and global consumption of cephalosporins and carbapenems between 2000 and 2015, using data from ResistanceMap. The results showed that the temporal trend of the proportion of tIS*26*‐bU^β‐lactam^‐positive strains was significantly associated with the consumption of cephalosporins or carbapenems in more than half (9/14 = 64.3%) of the countries (Table S4). The dynamics of the consumption of cephalosporins correlated with the proportion of tIS*26*‐bU^β‐lactam^ positive strains in Brazil, China, Germany, India, and the United States, while the consumption of carbapenems correlated with transposon dynamics in Australia, Canada, China, Denmark, India, and South Korea. These results suggest that the rapid spread of ARGs mediated by tIS*26*‐bUs could be promoted by increased consumption of antibiotics.

### IS*26*‐bUs exhibit a nonspecific target site preference

**Figure 4:**
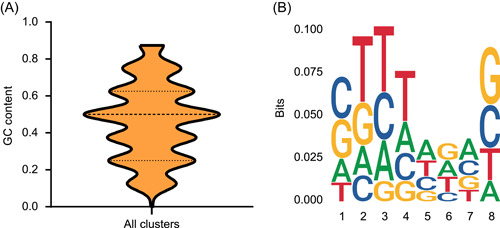
The features of IS*26* target site. (A) The GC content of IS*26* target sites. (B) The consensus sequence of IS*26* target sites.

To identify the reason for the frequent horizontal gene transfer of IS*26*‐bUs, we analyzed the characteristics of the 8‐bp direct repeat (DR) sequences generated by IS*26* insertion to determine whether the IS*26* elements exhibit a preference for target DNA sequences. A total of 226 8‐bp DR sequences of IS*26* elements were obtained. We first classified the sequences into clusters using CD‐HIT (nucleotide similarity of 100%), and 168 clusters were obtained, indicating the diversity of the DR sequences. We then calculated the mean GC content of the 8‐bp DR sequences and found that the value varied dramatically, from 0% to 87.5% (Figure 4A), suggesting that there was no obvious GC content preference of the target DNA sequences of IS*26*. We also analyzed sequence conservation of the nucleotides of the 8‐bp sequences and found a slight bias toward thymine at base positions 2–4 but no significant preference (Figure 4B). The information content of the 226 8‐bp DR sequences at any given position in the consensus sequence was lower than 0.1 bit, suggesting low conservation. Moreover, the emergence odds of four bases at any given position appeared random (range: 11.9%–39.9%; mean of 25%; standard deviation of 1%), indicating that the probability of occurrence of the four bases at each position is almost equal. Accordingly, we can conclude that IS*26*‐bUs insert into DNA sequences without preference.

### Unexpected rapid reaction to high antibiotic pressure of single IS*26*

**Figure 5:**
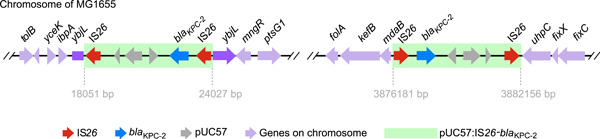
The genomic position of the two random insertions of pUC57:IS*26*‐*bla*_KPC‐2_ in the chromosome of MG1655.

During data analysis, we noticed that there were some strains carrying only one IS*26* gene (single‐copy) in the genome (located on plasmids or chromosomes). To explore the role of single‐copy IS*26* element in the dissemination of antimicrobial resistance, we constructed a plasmid (pUC57:IS*26*‐*bla*_KPC_) carrying an IS*26* element, an aminoglycoside resistance gene (*ampR*), and a carbapenem resistance gene (*bla*_KPC_) and introduced it into *E. coli* MG1655. Test cell cultures were exposed to antibiotic pressure (the concentration of carbapenem gradually increased from 1 to 256 μg/ml, doubled per inoculation), and control cells were cultured in a medium without antibiotics. We subsequently performed nanopore whole‐genome sequencing of the entire cell culture that survived under the highest carbapenem concentration (256 μg/ml) to detect the integration mediated by a single IS*26* and determine the effect of antibiotic pressure. Notably, the plasmid (pUC57:IS*26*‐*bla*_KPC‐2_) was observed randomly inserted into the chromosome of *E. coli* MG1655 cells challenged with carbapenem (Figure 5), demonstrating the mediation role of IS*26* during the insertion. The plasmid was inserted into two sites of the chromosome and was flanked by two IS*26* elements, thereby generating a new IS*26*‐bounded transposon carrying two ARGs, *ampR* and *bla*_KPC_ (Figure 5 and Table S5). One insertion site of the composite transposon was within the *ybiL* gene, resulting in a truncated gene; the other was in a noncoding region between the *mdaB* and *uhpC* genes. These results indicate that a single IS*26* can prompt random formation of ARG‐carried composite transposons, effectively increasing resistant levels under high antibiotic pressure. We also compared the hosts of tIS*26*‐bUs‐ARGs to those of IS*26* and detected a larger host range for IS*26* than for tIS*26*‐bUs‐ARGs. In total, 30 species belonging to 11 genera, three families, and two orders carried IS*26*, but did not contain tIS*26*‐bUs‐ARGs (Figure S4); this shows the potential for ARGs to reach new hosts mediated by IS*26*.

## DISCUSSION

It is well known that IS*26* mediates the transfer of many ARGs; however, the preference of ARGs carried by IS*26*, the phylogenetic distribution of the hosts, and the temporal dynamics of IS*26* remain elusive. In this study, we aimed to answer these questions and shed light on the dissemination of antibiotic resistance mediated by IS*26*. First, we demonstrated that 124 ARG types conferring resistance to 12 antibiotic classes were frequently carried by tIS*26*‐bUs. Other studies have shown that IS*Aba1* and its transposon were mainly responsible for the movement of the *bla*_OXA_ carbapenemase gene^13^, and 60 ARG genotypes and nine ARG phenotypes were detected on class 1 integrons^14^. Compared with these mobile elements, IS*26* carried more diverse and greater numbers of ARGs. Consistent with other studied mobile elements, tIS*26*‐bUs were widely distributed in Gram‐negative bacteria upon host analysis. In addition, tIS*26*‐bUs‐ARGs were predominantly found in clinically associated pathogens of *Gammaproteobacteria*, which was consistent with previous studies^10^, ^15^, ^16^. Notably, the average contribution rate of IS*26* to ARGs was 41.2%, and the contribution rate to some genes even reached 100%. These results suggest that IS*26* plays an important role in the spread of ARGs in bacteria, especially clinical pathogens.

Bacterial antibiotic resistance is becoming an increasingly serious problem and often occurs due to the acquisition of resistance genes by previously susceptible bacteria^17^. Our study revealed that tIS*26*‐bUs‐ARGs‐positive strains are widely spreading in bacterial populations and that this trend is increasing over time. This implies that IS*26* is closely associated with the development of bacterial antibiotic resistance. Tn*4401* was considered the origin of *bla*_KPC_ gene mobilization^18^; however, IS*26*‐based transposons, as a novel genetic environment for *bla*_KPC_, are increasingly being reported worldwide, especially in China^19^, ^20^, suggesting a trend for IS*26* to become a major genetic element for the acquisition and widespread dissemination of the *bla*_KPC_ gene. Moreover, the temporal relationship of tIS*26*‐bUs‐ARGs‐positive strains was closely related to the consumption of cephalosporins or carbapenems, indicating that IS*26* can respond to antibiotic pressure. Therefore, antibiotic usage should be strictly regulated and controlled to reduce the spread of antibiotic resistance.

Generally, the insertion of ISs shows target site preferences: for example, IS*1* prefers AT‐rich regions for insertion^21^, while IS*21*, IS*30*, and IS*911* all insert close to sequences that resemble their own inverted repeat (IR)^22^, ^23^, ^24^. However, the target sits of IS*26* was found with no obvious coincidence in a comparison of 14 insertion sites^25^. In the current analysis based on all the 2656 publicly available tIS*26*‐bUs, we further confirmed that IS*26* displayed a preference for random insertion, and thus has more opportunities for insertion. Moreover, previous research demonstrated the movement of the translocatable units formed by a single IS*26* ^26^. Consistent with this, our study also found that the introduction of a single copy of IS*26* can lead to the formation of IS*26*‐bounded transposons and the movement of flanking genes to rapidly respond to high antibiotic pressure. Notably, in our previous study, the transfer of the tIS*26*‐bU‐*bla*_KPC‐2_ among two clinical *E. coli* strains had been observed^10^. These observations illustrate the strong transfer potential of IS*26* during the mediation of antibiotic resistance and demonstrate the high risk of tIS*26*‐bU in horizontal gene transfer and the spread of ARGs.

Even though the analyzed genomes retrieved from the NCBI RefSeq database do not represent the entire range of genomes, they served as appropriate representatives of the microbes of interest for the current study. Using IS*26* as a mobile element model, we shed light on the relationship between ISs and ARGs and firmly established the critical role of ISs in the dissemination of antibiotic resistance. Given the significant contribution of IS*26* to the mediation of ARGs, especially in clinical pathogens, and the relationship observed with antibiotic usage, we strongly suggest that IS*26* be used as an indicator for monitoring antibiotic resistance in clinical practice.

## MATERIALS AND METHODS

### Bacterial genome preparation

A total of 12,142 complete bacterial genomes from the NCBI RefSeq genome database (https://ftp.ncbi.nlm.nih.gov/genomes/refseq/bacteria/) were downloaded on March 11, 2021. The isolation time, isolation source, country or region, and taxonomic lineages of the genome's host were retrieved from their GenBank files. The isolation, sequencing, submission, and data releasing of a strain would last at least 3–5 years. Thus, there is a latency between bacterial genome submission and strain isolation. According to the isolation time, we noticed that for the 12,142 downloaded genomes, there are too few genomes isolated after the year 2018. Therefore, we only used strains isolated before 2018 to ensure a complete data set for the analysis of temporal trends of IS*26*, avoiding sample bias. The isolation source was further divided into human, animal, environmental, other sources, and unknown sources. These data are summarized in Table S3.

### Data collection for antibiotic use

Data indicating the time when specific antibiotics received FDA approval for human application were retrieved from the study of Durand^27^. Consumption data for cephalosporins and carbapenems of the countries and regions involved in our study were collected from ResistanceMap (https://resistancemap.cddep.org/AntibioticResistance.php), as previously described^28^.

### Extraction and annotation of IS*26*‐bounded transposons and related sequences

The nucleotide sequence of the IS*26* element was downloaded from the ISfinder database^29^. A BLASTN search for the IS*26* sequence in complete bacterial genomes within the NCBI database was performed with cut‐offs of at least 95% nucleotide similarity and 95% IS*26* sequence coverage to annotate the IS*26* element. Based on the genomic position of pairwise alignment of IS*26* in replicons identified by BLASTN, DNA sequences flanked by two copies of IS*26*, defined as the IS*26*‐bUs, were extracted. Then, we assigned the IS*26*‐bU sequences to different clusters using CD‐HIT^30^, according to a nucleotide similarity of 99%. Next, we extracted the 5‐kb upstream and downstream genomic sequences surrounding the IS*26*‐bU and again clustered these sequences using CD‐HIT at a nucleotide similarity of 99%. We checked and classified the IS*26*‐bU located in different genomic environments as translocatable, defined as the translocatable tIS*26*‐bU. Moreover, the ARGs were annotated by using ResFinder v4.1.0^31^ for these tIS26‐bUs to obtain the tIS*26*‐bUs‐ARGs. The contribution of tIS*26*‐bU to ARGs was calculated by dividing the number of the ARGs on the tIS*26*‐bU by the number of ARGs annotated in the NCBI complete genome, multiplied by 100, as shown in Equation (1).

(1) $${\text{Contribution rate}}_{{\text{ARG}}_{i}}=\frac{\text{Number}\;\text{of}\;{\text{ARG}}_{i}\;\text{on tIS}26‐\text{bUs}}{\text{Number}\;\text{of}\;{\text{ARG}}_{i}\;\text{in}\;\text{NCBI complete genome}}\times 100$$

### Construction of phylogenetic tree

The core genes of the tIS*26*‐bUs‐ARGs‐positive strains were constructed through the Roary v3.13.3^32^ based on the protein annotation files of all the strains. Then, the concatenated sequence of the core genes was used to construct the phylogenetic tree by using RAxML^33^. The tree file was further illustrated on the iTol (https://itol.embl.de/itol.cgi).

### Statistical analysis

The proportion of tIS*26*‐bUs‐ARGs over time was normalized by dividing the genome number of tIS*26*‐bUs‐ARGs in a given year by the total number of genomes in the database for that year. Then, linear regression analysis to determine the changing trend of proportion of tIS*26*‐bUs‐ARGs‐positive strains along with the isolation time thereof was performed using GraphPad Prism software for Windows (ver. 8.0.1; GraphPad Software Inc.). For trend analysis, the Cochran–Armitage and Mann–Kendall tests were performed using the “DescTools” (ver. 0.99.44) and “trend” (ver. 1.1.4) packages in R software (ver. 4.1.2). The scipy module (ver. 1.5.4) in Python (ver. 3.6.13) was used to calculate the Spearman's correlation coefficient. The “sampling” package (ver. 2.9) for R (ver. 3.5.1) was used for the random sampling of genomes each year.

### Target site sequence analysis

The 8‐bp DR sequences located on each side of the IS*26* elements were extracted from the genomes. Then, the obtained 8‐bp DR sequences were clustered using CD‐HIT (100% nucleotide identity and 100% coverage)^30^. The ggplot2 (ver. 3.3.3) and ggseqlogo (ver. 0.1) in R (ver. 3.5.1) were used to analyze the GC content and nucleotide sequence conservation of target sequences, respectively.

### In vitro experiments using a single‐copy IS*26* strain

The construction and validation of the plasmid pUC57:IS*26*‐*bla*_KPC‐2_ (5149 bp) were performed as previously described^12^. Briefly, the pUC57:IS*26*‐*bla*_KPC‐2_ plasmid was constructed by assembling three fragments, including an IS*26* gene, a *bla*_KPC‐2_ gene, and a pUC57 vector, using an NEBuilder HiFi DNA assembly cloning kit. Then, pUC57:IS*26*‐*bla*_KPC‐2_ was transformed into *E. coli* MG1655 and coated onto LB agar plates containing kanamycin (50 μg/ml) and meropenem (2 μg/ml) for the selection of transformant. A single colony was picked from the transformation plate and inoculated with 200 μl of LB media in a 96‐cell plate and incubated at 37°C to create a starter culture. Bacterial cells from the starter culture were then separately transferred to 200 μl of LB media containing meropenem or LB media without antibiotic (as control) per 12 h. The concentration of meropenem was gradiently increased by two times during each transfer, starting at 1 μg/ml, and doubled up to 256 μg/ml. In detail, the bacterial cells from the starter culture were inoculated to LB media with a carbapenem of 1 μg/ml. Then, the culture was further inoculated every 12 h, and the concentration of carbapenem was doubled each time until up to 256 μg/ml carbapenem. All the cultures were incubated at 37°C. Three biological replicates were performed. Then, the complete genome sequences for the whole culture treated with 256 μg/ml were obtained through the PromethION sequencing platform. To detect the insertion site of the pUC57:IS*26*‐*bla*_KPC‐2_ plasmid, homogeneous search for IS*26*‐*bla*_KPC‐2_ against the complete genome we sequenced above was performed by BLASTN.

## AUTHOR CONTRIBUTIONS

**Na Tang**: Data curation (equal); formal analysis (equal); methodology (equal); software (equal); writing—original draft (equal). **Dawei Wei**: Validation (equal); visualization (equal). **Yuan Zeng**: Methodology (equal); software (equal). **Gang Zhang**: Investigation (equal); project administration (equal). **Chao Wang**: Methodology (equal); resources (equal). **Jie Feng**: Conceptualization (equal); investigation (equal); supervision (equal); writing—original draft (equal); writing—review and editing (equal). **Yuqin Song**: Formal analysis (equal); funding acquisition (equal); methodology (equal); software (equal); validation (equal); visualization (equal); writing—original draft (equal); writing—review and editing (equal).

## ETHICS STATEMENT

No trials on humans or animals were involved in this study.

## CONFLICT OF INTERESTS

The authors declare no conflict of interests.

## Supporting information

Supporting information.

**Figure S1. Host distribution of translocatable IS*26*‐bounded units carrying ARGs (tIS*26*‐bU‐ARGs)**. Circle and hexagon sizes are proportional to the number of genomes with tIS*26*‐bUs‐ARGs on the species level, and each color represents the different taxonomies.

**Figure S2. Schematic of the structure of the transposons**.

**Figure S3. The result of the proportion of translocatable IS*26*‐bounded units with ARGs (tIS*26*‐bU‐ARGs) positive strains in sequenced strains treated with different analysis methods over time**. (A) The proportion of tIS*26*‐bUs‐ARGs positive strains in sequenced strains from different isolation sources. (B) The proportion of tIS*26*‐bUs‐ARGs positive strains in sequenced strains over time. (C) The differences in the proportion of tIS*26*‐bUs‐ARGs‐positive strains from different isolation sources.

Figure S4. The future expansion potential of antibiotic resistance into the new host at different taxonomy levels.

## Data Availability

The complete genome sequence for one culture is available under the NCBI GeneBank database (accession no. PRJNA889197).
