# Supplementary figures and images for: Understanding the rapid spread of antimicrobial resistance genes mediated by IS26
Source: mLife. 2024 Mar 18;3(1):101–9. doi: 10.1002/mlf2.12114 (PMC11139202; doi:10.1002/mlf2.12114)

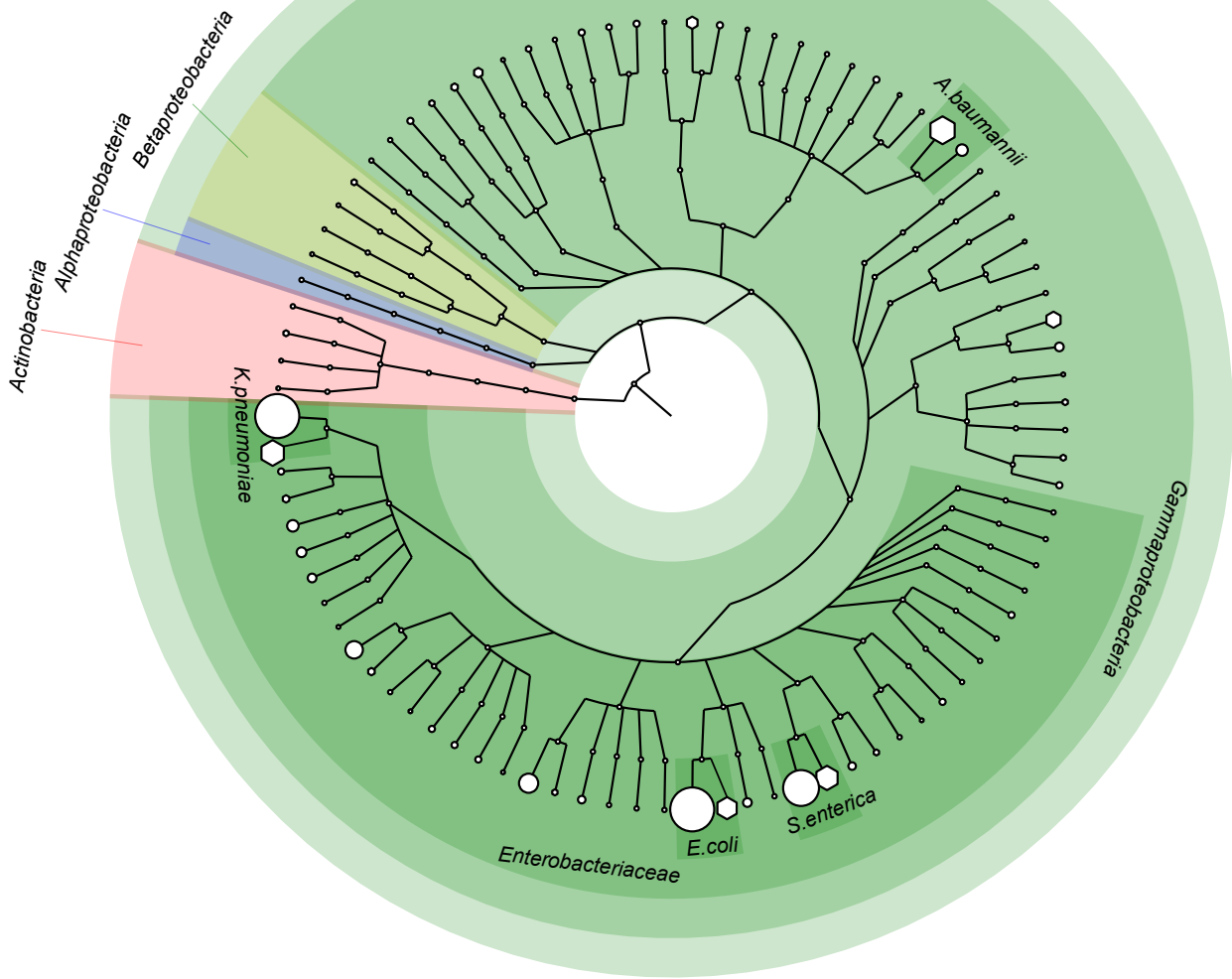

Supplement: Supplementary file 2 — Figure S1. Host distribution of translocatable IS26‐bounded units carrying ARGs (tIS26‐bU‐ARGs). Circle and hexagon sizes are proportional to the number of genomes with tIS26‐bUs‐ARGs on the species level, and each color represents the different taxonomies. [file MLF2-3-101-s001.pdf]

(A)

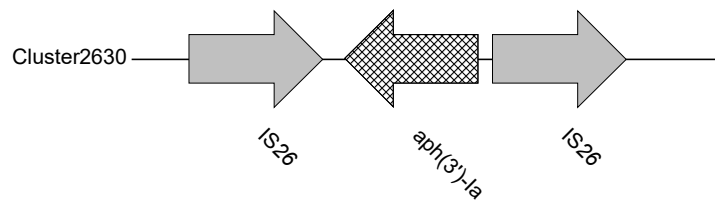

(B)

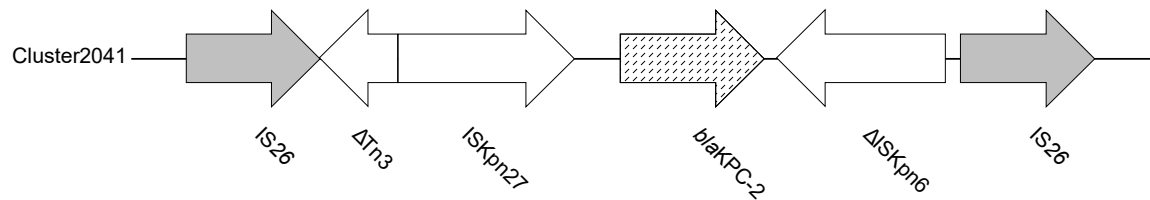

(C)

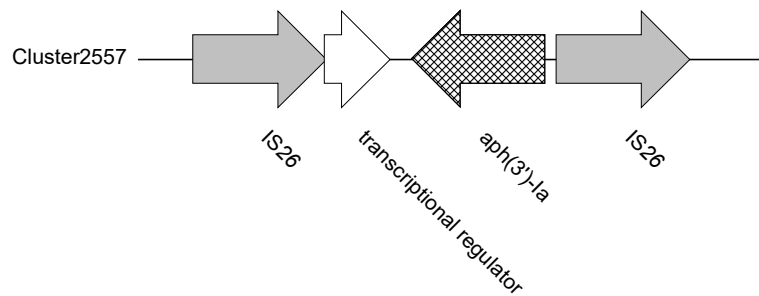

Supplement: Supplementary file 3 — Figure S2. Schematic of the structure of the transposons. [file MLF2-3-101-s005.pdf]

(A)

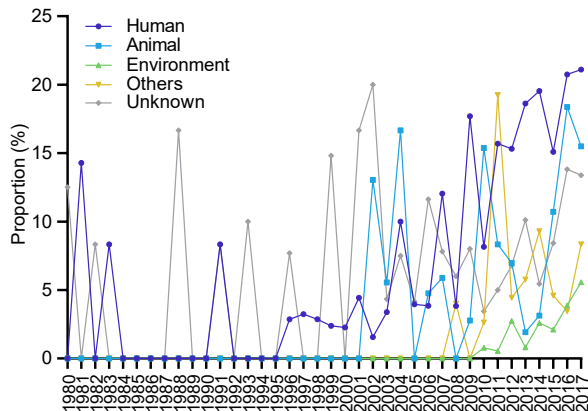

(B)

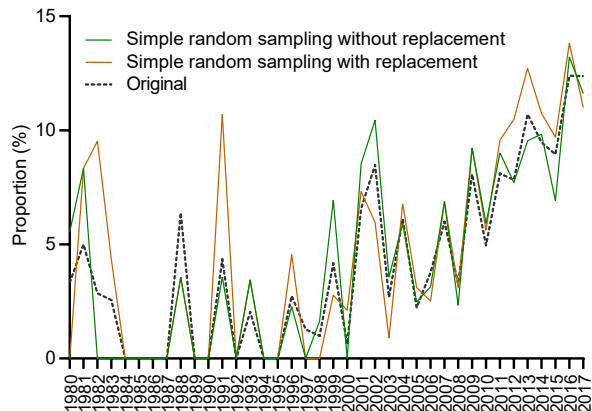

(C)

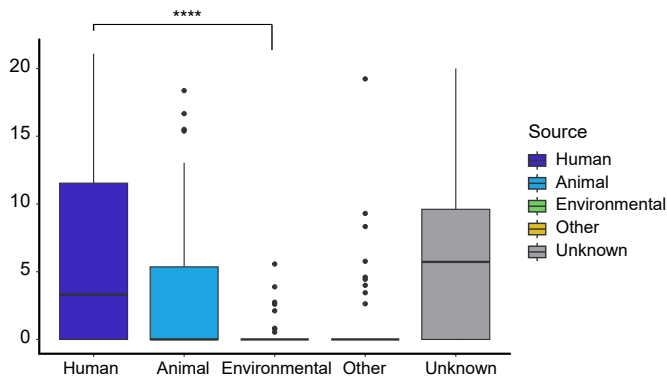

Supplement: Supplementary file 4 — Figure S3. The result of the proportion of translocatable IS26‐bounded units with ARGs (tIS26‐bU‐ARGs) positive strains in sequenced strains treated with different analysis methods over time. (A) The proportion of tIS26‐bUs‐ARGs positive strains in sequenced strains from different isolation sources. (B) The proportion of tIS26‐bUs‐ARGs positive strains in sequenced strains over time. (C) The differences in the proportion of tIS26‐bUs‐ARGs‐positive strains from different isolation sources. [file MLF2-3-101-s003.pdf]

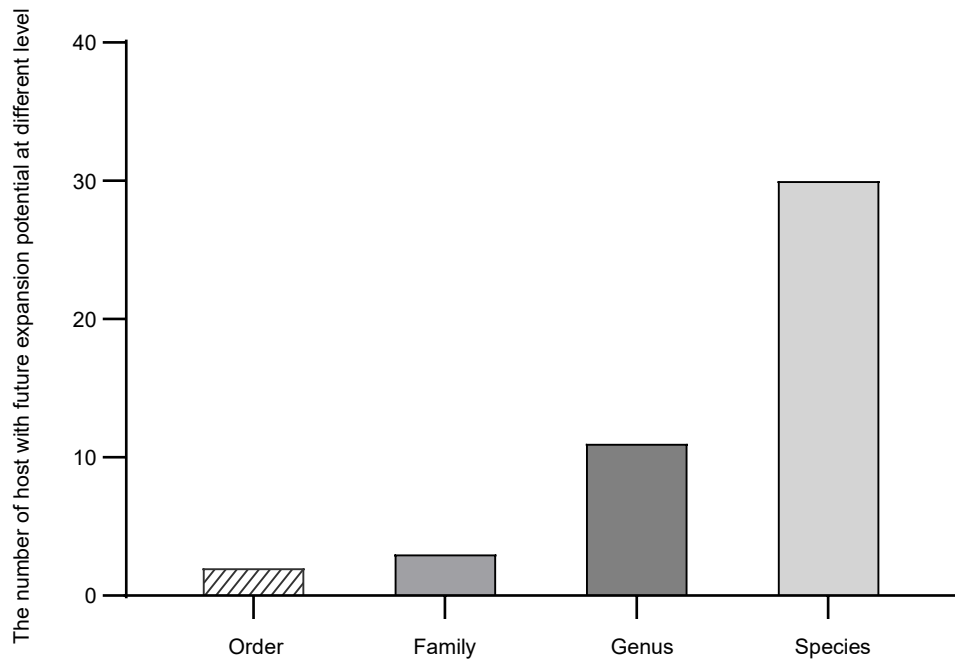

Supplement: Supplementary file 5 — Figure S4. The future expansion potential of antibiotic resistance into the new host at different taxonomy levels. [file MLF2-3-101-s002.pdf]
